# Ivermectin induces cell cycle arrest and caspase-dependent apoptosis in human urothelial carcinoma cells

**DOI:** 10.7150/ijms.76623

**Published:** 2022-09-11

**Authors:** Chun-Liang Tung, Wen-Ying Chao, Yi-Zhen Li, Cheng-Huang Shen, Pei-Wen Zhao, Shu-Hsin Chen, Tzu-Yun Wu, Ying-Ray Lee

**Affiliations:** 1 Department of Pathology, Ditmanson Medical Foundation Chia-Yi Christian Hospital, Chiayi, Taiwan.; 2 Department of Food Nutrition and Health Biotechnology, Asia University, Taichung, Taiwan.; 3 Department of Nursing, Min-Hwei College of Health Care Management, Tainan 73658, Taiwan.; 4 Department of Medical Research, Ditmanson Medical Foundation Chiayi Christian Hospital, Chiayi, Taiwan.; 5 Department of Urology, Ditmanson Medical Foundation Chia-Yi Christian Hospital, Chiayi, Taiwan.; 6 Department of Microbiology and Immunology, College of Medicine, Kaohsiung Medical University, Kaohsiung, Taiwan.; 7 Master of Science Program in Tropical Medicine, College of Medicine, Kaohsiung Medical University, Kaohsiung, Taiwan.

**Keywords:** bladder cancer, ivermectin, anticancer activity, apoptosis

## Abstract

Bladder carcinoma is one of the most common malignancies worldwide, and >90% of all bladder cancers are classified as urothelial carcinomas (UC). Surgery, radiotherapy, chemotherapy, targeted therapy, and immunotherapy are evidence-based treatments that are administered depending on the clinical stage of UC. All these treatments exhibited limited effects in cases of metastatic UC, and UC with specific location, invasiveness, and recurrence. Therefore, a new therapeutic strategy for UC is urgently needed.

Ivermectin, an avermectin derivative, has been reported to be effective against various parasites, and its pharmacokinetic and pharmacodynamic properties as well as safety are well understood in humans. Recently, ivermectin was shown to exhibit therapeutic benefits against various virus infections *in vitro*, and anticancer activity against various human cancer cells. This study aimed to investigate the anticancer effects of ivermectin in human UC cells. Ivermectin inhibited growth, regulated the cell cycle, and induced apoptosis in human UC cells. It also induced the activation of both extrinsic and intrinsic caspase-dependent apoptotic pathways. Further investigation revealed that ivermectin induced apoptosis in UC cells is mediated via c-Jun N-terminal kinase signaling. Herein, we demonstrated that ivermectin can be used as a new therapeutic agent for treating UC cells.

## Introduction

Urothelial carcinoma (UC), also known as transitional cell carcinoma, is one of the most common malignancies worldwide 1. Currently, therapeutic strategies such as surgery, radiotherapy, chemotherapy, targeted therapy, and immunotherapy are provided depending on the clinical stage of UC. However, the curative effects of these treatments are limited by the specific location, invasiveness, and recurrence of metastatic UC. The 5-year survival rate of patients with UC that has not yet spread out of the bladder is 70%; however, when the tumor extends outside the bladder or spreads to nearby lymph nodes, the 5-year survival rate decreases to <40%. Moreover, in patients with distant metastasis, the 5-year survival rate is 6% 2. Therefore, there is an urgent need for developing of a new therapeutic strategy for UC. However, considering the long process of developing novel drugs, the off-label use of current clinical agents is a good strategy for developing a new therapeutic agent for UC. In this study, we investigated the anti-UC activity of ivermectin in human UC cells.

Ivermectin, an avermectin derivative, is a broad-spectrum drug widely used against parasitic infections in humans including onchocerciasis, strongyloidiasis, ascariasis, cutaneous larva migrans, filariases, gnathostomiasis, trichuriasis, pediculosis and scabies, and its pharmacokinetic and pharmacodynamic properties as well as safety are well understood 3. Moreover, it exhibits antiviral activity against various RNA and DNA viruses 4. In addition, ivermectin has shown anticancer activity in various human cancers, including leukemia, melanoma, esophageal squamous cell carcinoma, glioma, and breast, ovarian, and colon cancers 5-7. Moreover, it has been demonstrated to inhibit tumor proliferation, metastasis, and angiogenesis in various cancer cells 8, as well as reverse drug resistance when combined with clinical chemotherapeutic agents 9. However, the molecular mechanism underlying ivermectin's anticancer effects have not been investigated in human UC cells.

Clinically, intravesical Bacillus Calmette-Guérin immunotherapy is used to decrease the progression of non-muscle-invasive bladder cancer; however, it may result in various side effects, such as chemical cystitis. Therefore, in contrast to chemotherapeutics administered orally or through intravenous injection in various cancers, intravesical injection is allowed in human UCs. In this study, we used two human UC cell lines, T24 and RT4, to investigate the anti-UC activity of ivermectin. We found that ivermectin could suppress cellular proliferation and induce cell cycle arrest and apoptosis. Furthermore, ivermectin mediated caspase-dependent apoptosis. Finally, we identified a novel molecular mechanism underlying the apoptosis induction of UC cells by ivermectin.

## Material and methods

### Cell culture and treatment

Human UC cell lines T24 and RT4, were procured from the Bioresource Collection and Research Center (Hsinchu, Taiwan). Both cells were cultured with McCoy's 5A medium supplemented with 10% fetal bovine serum and preserved under 5% CO_2_ at atmosphere at 37 °C. Ivermectin was purchased from Sigma-Aldrich (St. Louis, MO, USA) and dissolved in dimethyl sulfoxide (DMSO; Sigma-Aldrich) to prepare the stock and further experiments. As vehicle control, the cells were treated with 1% DMSO.

### Cell proliferation assay

The cells were seeded into 96-well culture plates (5 × 10^3^ cells/well) and incubated with medium only (containing 1% DMSO as the negative control) or with medium containing ivermectin. Cell viability was determined using the Cell Counting Kit-8 (CCK-8) (Sigma-Aldrich), as previously described 10.

### Cell cycle analysis

Cells (1 × 10^5^ cells/dish) were treated with DMSO or ivermectin for the corresponding time after starvation, washed once with phosphate-buffered saline, and finally fixed with 100% methanol. The fixed cells were incubated with RNase (10 mg/mL; Sigma-Aldrich) and propidium iodide (PI; 1 mg/mL; Sigma-Aldrich) in the dark for 30 min. The DNA content of the cells was assessed using FACScan (Becton Dickinson, San Diego, CA, USA) equipped with the ModFit LT 3.3 software. In addition, the cell cycle markers were examined by Western blotting with specific antibodies including p21 (#2947, 1:1000, Cell Signaling; Danvers, MA, USA), CDK2 (#2546, 1:1000, Cell Signaling), CDK4 (#2906, 1:1000, Cell Signaling), and cyclin D1 (#2922, 1:1000, Cell Signaling).

### Cell apoptosis assay

Cells (1 × 10^6^ cells/dish) were treated with ivermectin or DMSO. Cell apoptosis was evaluated using the Annexin-V-FITC apoptosis detection kit (Strong Biotech, Taipei, Taiwan). After treatment, cells were incubated with FITC-labeled annexin-V and PI for 15 min at room temperature, and the intensity of annexin-V or PI fluorescence was determined using FACScan (Becton Dickinson); 10,000 cells were examined per sample.

To investigate the mechanisms underlying ivermectin-mediated apoptosis, the activation of caspase-3, -8, -9 (#9662, #9746, #9502, Cell Signaling; with 1:1000 dilution), Bid (GTX60429, 1:1000, GeneTex, Hsinchu City, Taiwan), Bcl-xL (#2762, Cell Signaling) and poly (ADP-ribose) polymerase (PARP; #9542, Cell Signaling; with 1:1000 dilution) were assessed using western blotting. To confirm whether caspase activation is involved in ivermectin-mediated cellular apoptosis, the pan-caspase inhibitor, Z-VAD-fmk (BioVision, Mountain View, CA, USA), was used and cellular apoptosis was assessed using FACScan after double staining with FITC-labeled annexin-V and PI. The GAPDH (GTX100118, 1:200000, GeneTex) was determined as a loading control in the Western blotting.

In addition, to examine the upstream signaling pathways involved in ivermectin-mediated anti-UC effects, the specific antibodies including Erk (#4696, 1:1000, Cell Signaling), phosphor-Erk (#9106, 1:1000, Cell Signaling), p38 (#9212, 1:1000, Cell Signaling), phosphor-p38 (#9211, 1:1000, Cell Signaling), JNK (#9252, 1:1000, Cell Signaling), phosphor-JNK (#9251, 1:1000, Cell Signaling), Akt (#9272, 1:1000, Cell Signaling), and phosphor-Akt (#9271, 1:1000, Cell Signaling) were used to detection the protein expressions using western blotting. Moreover, JNK and ERK inhibitors (SP600125 and PD98059) were purchased from LC Laboratories (Woburn, MA, USA) and were used to increase the suppression of JNK or ERK activation. Cell viability and apoptosis were determined using CCK-8 assay and FACScan as previously described 11.

### Mitochondrial membrane potential assay

To evaluate the mitochondrial membrane potential (MMP), cells (1 ×10^6^ cells/dish) were treated with ivermectin or DMSO for the indicated time, following which MMP was detected using FACScan after rhodamine-123 fluorescent (2 mM; Sigma-Aldrich) staining for 2 h.

### Statistical analysis

Data are presented as the mean ± SD of separate experiments. Differences between the test and control groups were analyzed using one-way ANOVA and Fisher's least significant difference test. A *P* value of < 0.05 was considered statistically significant in all tests.

## Results

### Ivermectin suppresses cell proliferation in human UC cells

**Figure 1:**
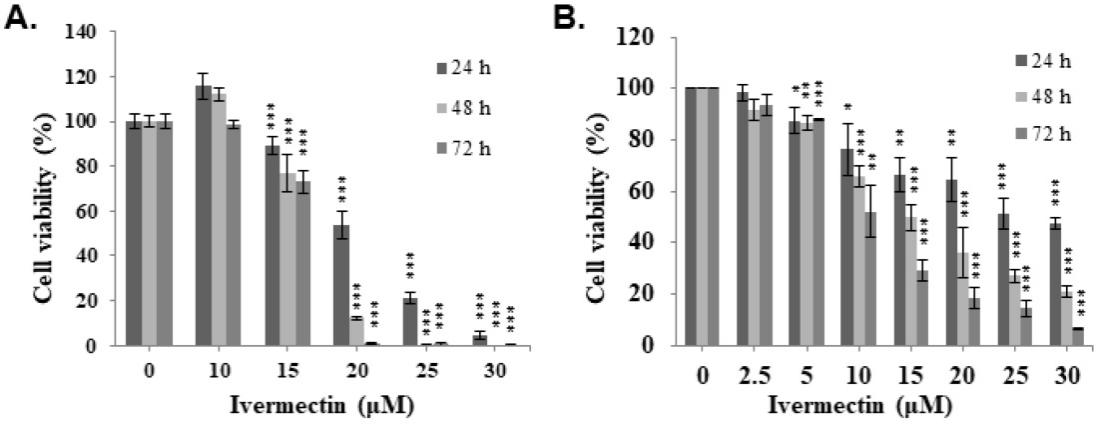
** Ivermectin inhibits cell viability of human urothelial carcinoma cells. (A)** T24 and **(B)** RT4 cells were treated with various ivermectin doses, and cellular viability was assessed via CCK-8 assay. DMSO was used as negative control. The results are shown as the mean ± S.D (n = 6) of three independent experiments. *Comparing with the control group. * *p* < 0.05, ** *p* < 0.01, *** *p* < 0.001.

To evaluate the anti-UC activity of ivermectin, two human UC cell lines, T24 and RT4, were treated with ivermectin, and cell viability was examined using CCK-8 assay. The results revealed that ivermectin inhibited cell proliferation in these cells in a dose- and time-dependent manner (Figure 1). The IC50s in T24 cells were 20.5, 17.4 and 16.6 μM, and in RT4 cells were 26.7, 14.9, and 10.0 μM at 24, 48, and 72 h post-incubation, respectively. These findings suggested that T24 cells were sensitive to ivermectin treatment at short term administration, whereas RT4 cells were sensitive to this drug at long term administration.

### Ivermectin induces cell cycle arrest at the G1 phase in human UC cells

**Figure 2:**
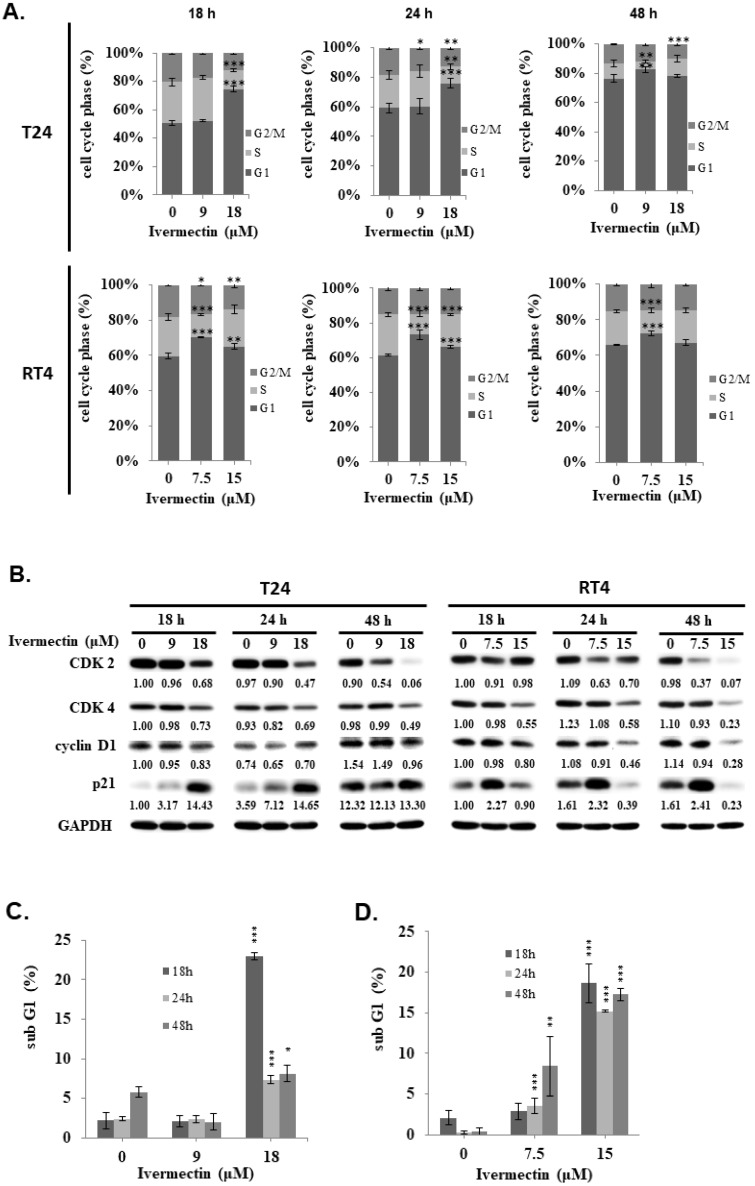
** Ivermectin induces cell cycle arrest and sub-G1 population increased in human urothelial carcinoma cells. (A)** T24 and RT4 cells were treated with ivermectin, and the cell cycle phase was determined via flow cytometry. **(B)** Cell cycle markers were examined by western blotting in cells treated with ivermectin. The sub-G1 population in **(C)** T24 and **(D)** RT4 cells was determined with flow cytometry. DMSO was used as negative control. Three independent experiments each for western blotting and flow cytometry were conducted; a representative western blotting is shown. *Compared with the control group. * *p* < 0.05, ** *p* < 0.01, *** *p* < 0.001.

To investigate the mechanism underlying the anti-UC activity of ivermectin, a cell cycle analysis was performed. T24 and RT4 cells were treated with ivermectin, and flow cytometry was used to estimate the percentages of cell population in the different phases of the cell cycle. Ivermectin induced cell cycle arrest at the G1 phase (Figure 2A and Supplementary Figure 1). Furthermore, cell cycle markers, including p21, CDK2, CDK4, and cyclin D1, were examined in cells treated with ivermectin, which revealed increased p21 and decreased CDK2, CDK4, and cyclin D1 expression in these cells (Figure 2B). These findings demonstrated that ivermectin induces cell cycle arrest at the G1 phase in T24 and RT4 cells. In addition, we found a significantly sub-G1 population increased in T24 and RT4 cells under ivermectin treatment. However, RT4 cells exerted higher sub-G1 population compared with T24 cells when treated with ivermectin (Figure 2C and 2D), suggesting that ivermectin significantly increases apoptosis in RT4 cells.

### Ivermectin induces caspase-dependent apoptosis in human UC cells

**Figure 3:**
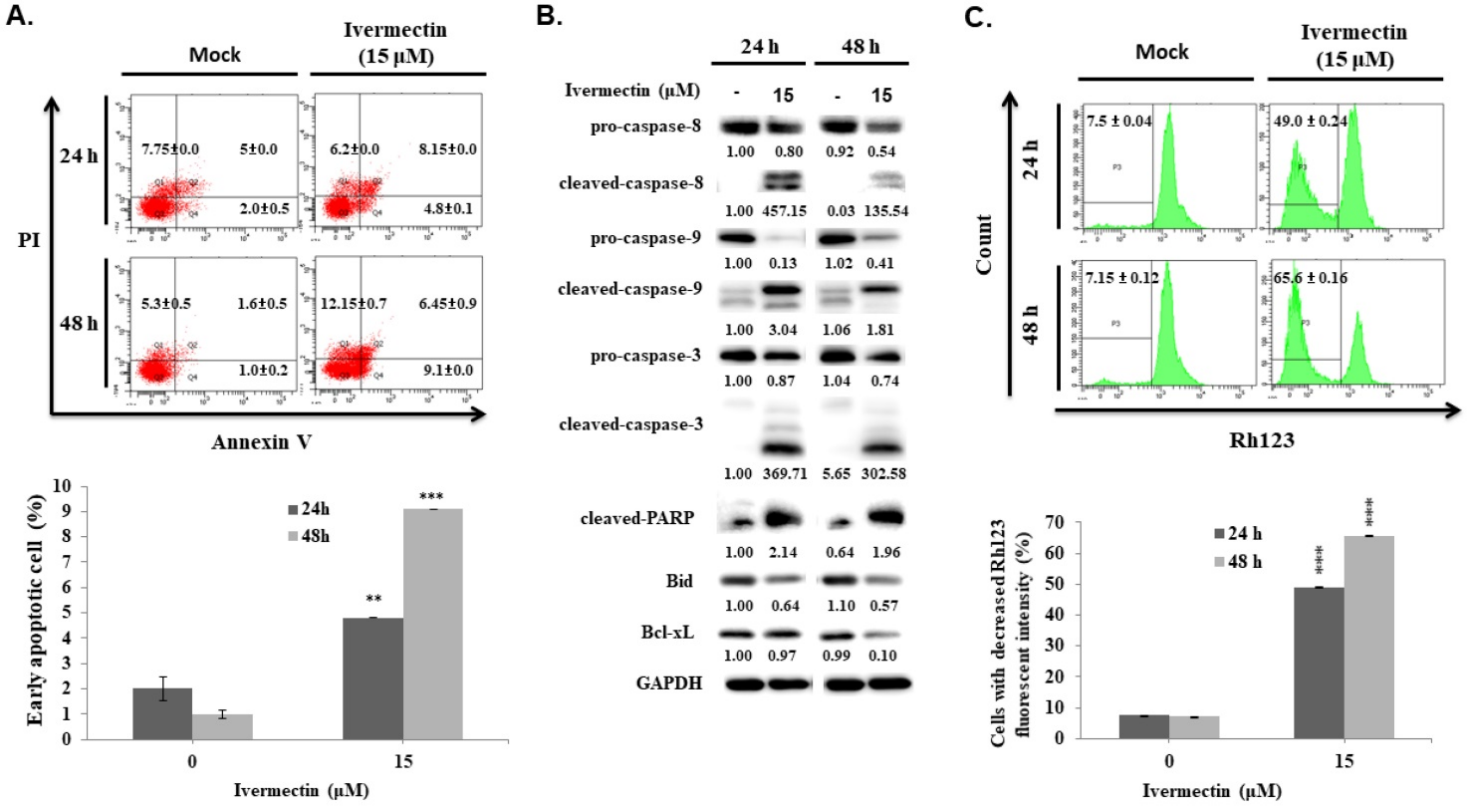
** Ivermectin induces apoptosis in human urothelial carcinoma cells.** RT4 cells were treated with ivermectin, **(A)** and early apoptotic cells were examined via flow cytometry. **(B)** Caspase-3, -8, -9, PARP, Bid, and Bcl-xL protein expression was assessed using western blotting in cells treated with ivermectin. **(C)** MMP was evaluated via flow cytometry in RT4 cells after treatment with ivermectin. DMSO was used as negative control. Three independent experiments each were conducted for flow cytometry and western blotting; Western blots and flow cytometry dot plots of a representative experiment are shown. *Compared with the control group. ** *p* < 0.01, *** *p* < 0.001.

We found a significantly sub-G1 population increased in RT4 cells under coincubation with ivermectin. To determine whether apoptosis is involved in the anti-human UC activity of ivermectin, cellular apoptosis was examined via flow cytometry in cells treated with ivermectin. The results showed that ivermectin significantly increased early apoptosis in RT4 cells in a time-dependent manner (Figure 3A). Further investigation on the underlying mechanisms of ivermectin-mediated apoptosis in RT4 cells revealed that RARP and other upstream factors, including caspase -8, -9, and -3, were activated, whereas Bid and Bcl-xL expression had decreased (Figure 3B). These findings suggested that ivermectin could upregulate both intrinsic and extrinsic caspase apoptotic pathways in RT4 cells. To confirm that the intrinsic pathway was involved in ivermectin-induced apoptosis, MMP was determined using rhodamine 123 staining in RT4 cells treated with ivermectin. A significant dose-dependent change in MMP was observed in RT4 cells (Figure 3C). In addition, ivermectin-mediated caspase-dependent apoptosis was also observed in T24 cells (Supplementary Figure 2).

**Figure 4:**
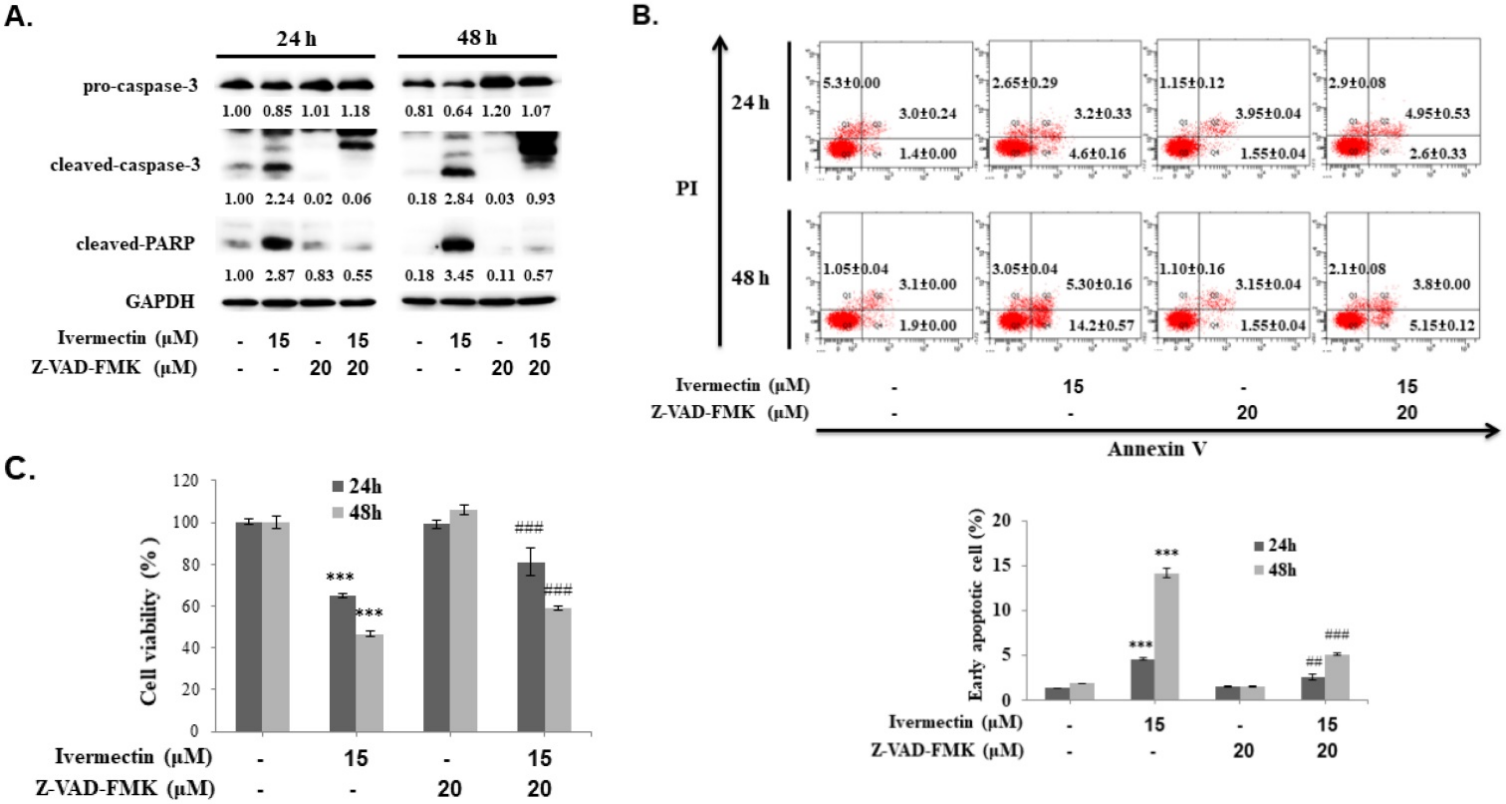
** Ivermectin-induced apoptosis in human urothelial carcinoma cells was caspase-dependent.** RT4 cells were pre-incubated with Z-VAD-FMK before ivermectin treatment, **(A)** caspase-3 and PARP expression was examined using western blotting, and **(B)** early apoptotic cells were quantified via flow cytometry. **(C)** Total cellular viability was measured via CCK-8 assay. DMSO was used as negative control. Three independent experiments each were conducted for flow cytometry and western blotting; western blots and flow cytometry dot plots of a representative experiment are shown. *Compared with the control group, and # compared with the ivermectin treatment group. *** and ### p < 0.001.

To confirm that the anti-UC activity of ivermectin involves caspase-dependent apoptosis, cells were pretreated with a pan-caspase inhibitor, Z-VAD-FMK, following which caspase-3 and PARP expression as well as early cellular apoptosis and survival were assessed after ivermectin treatment. As shown in Figure 4A, ivermectin-mediated caspase-3 and PARP activation could be suppressed by Z-VAD-FMK pretreatment. Moreover, under these conditions, ivermectin-mediated early cellular apoptosis was significantly reduced (Figure 4B). Total cell survival was also reversed in cells pretreated with Z-VAD-FMK (Figure 4C). These findings demonstrated that ivermectin treatment induced caspase-dependent apoptosis in human UC cells, thereby exhibiting antitumor activity.

### Ivermectin mediates cell apoptosis through the JNK pathway

**Figure 5:**
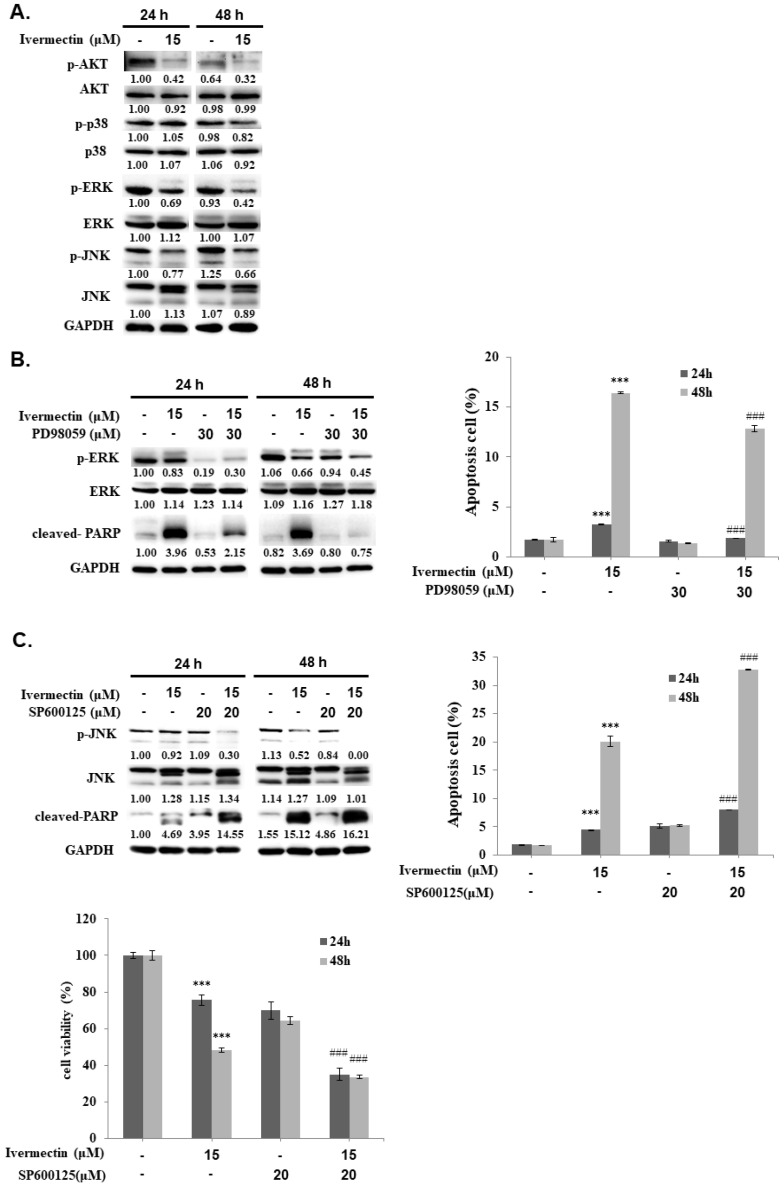
** Ivermectin-mediated apoptosis through suppression of JNK signaling. (A)** RT4 cells were treated with ivermectin, and p38, ERK, and JNK expressions were assessed using western blotting. **(B)** PD98059 and **(C)** SP600125 were used to inhibit ERK or JNK activation, and ERK and JNK expression and activation were confirmed using western blotting. Total apoptotic cells and total cell survival were evaluated via flow cytometry and CCK-8 assay. DMSO was used as negative control. Three independent experiments each were conducted for flow cytometry and western blotting; Western blots and flow cytometry of a representative experiment are shown. *Compared with the control group, and # compared with the ivermectin treated group. *** and ### *p* < 0.001.

The mitogen-activated protein kinase (MAPK) signaling pathway plays an important role in tumor cell proliferation, differentiation, and survival. Moreover, ivermectin-attenuated phosphorylation of JNK, ERK, and p38 MAPK (p38) has been previously reported 9, 12, 13. To further investigate the upstream signaling pathways involved in ivermectin-induced cellular apoptosis, western blotting was performed to determine the expression of p38, ERK, and JNK pathways. Ivermectin significantly suppressed activation of the ERK and JNK pathways but not that of the p38 pathway in RT4 cells (Figure 5A). Therefore, PD98059 was used to enhance ERK suppression in cells treated with ivermectin, and ERK and PARP activation were detected by western blotting. The results showed that PD98059 could suppress ERK activation in RT4 cells treated with ivermectin (Figure 5B). However, under this condition, no further increase in the cleavage of PARP was found, suggesting that the inhibition of ERK signaling was not the upstream mechanism of ivermectin-mediated apoptosis. Further research on cellular apoptosis using flow cytometry confirmed this finding (Figure 5B). In addition, SP600125 was used to suppress JNK activation, and activated JNK and PARP levels were detected using western blotting. The results showed that a combination treatment of ivermectin and SP600125 reduced JNK activation, thereby increasing PARP activation (Figure 5C). This finding suggested that JNK signaling was the upstream mechanism of ivermectin-mediated apoptosis in RT4 cells. Moreover, the combination treatment of ivermectin and SP600125 significantly increased anticancer activity, thus increasing apoptosis and reducing total cell viability (Figure 5C). Taken together, these findings demonstrated that ivermectin-induced apoptosis was mediated by JNK signaling.

## Discussion

Currently, in addition to surgery, tumor apoptosis induction is the most successful treatment for human cancers. However, in patients with cancer, surgery is limited in terms of the location of some cancers or large-scale recurrence. Therefore, chemotherapy is a beneficial approach, exerting excellent effects. However, heterogenous mutations, resulting in tumor resistance to chemotherapy is common in various human cancers. Therefore, there is an urgent need to develop various chemotherapeutic agents for human cancers. UC is one of the most critical malignancies that can metastasize into proximal or distal tissues through invasion and migration, thus resulting in substantial mortality rates. UC is the primary cause of health issues owing to its high recurrence rate and lack of treatment efficacy. Therefore, there is pressing need for novel therapeutic strategies for patients with UC. In this study, we demonstrated that ivermectin, a classical therapeutic agent against parasitic infection, exhibits effective anti-UC activity through cell cycle regulation and apoptosis induction. Further investigation demonstrated that ivermectin-induced UC cells apoptosis was mediated by JNK signaling pathway suppression and downstream caspase-dependent apoptosis.

Ivermectin is a polycyclic lactone pesticide derived from Streptomyces avermitilis and exerts a broad-spectrum effect against parasites. It also displayed an anti-viral activity in various viruses 4. Current research has demonstrated the potent effects of ivermectin against SARS-CoV-2 *in vitro*, regardless of strain and variant 14. However, a clinical study, in patients with COVID-19 infection who received ivermectin found no significant difference in the incidence of serious illness-related hospital admissions 15. In addition, ivermectin has been reported to show antitumor activity in various human cancers, including leukemia, melanoma, esophageal squamous cell carcinoma, glioma, and breast, ovarian, and colon cancers by increasing cell proliferation inhibition, cell cycle arrest, and cell apoptosis or autophagy 5-7. Ivermectin has been used for many years to treat parasitic infection, and despite exhibiting antitumor activity in several cancers, its antitumor effect on human UCs remains unclear. In this study, we demonstrated that treatment with ivermectin significantly suppressed human UC cell proliferation in a dose- and time-dependent manner (Figure. 1). This finding is consistent with that of previous studies 5-7. Notably, a previous study demonstrated that low ivermectin concentrations had no cytotoxic effect, whereas ivermectin at a concentration of ≥20 μM slightly inhibited the viability of normal cells after a 48 h exposure 6. Our findings showed that ivermectin's IC50 values at 48 h after treatment were 17.4 μM in T24 cells and 14.9 μM in RT4 cells, both of which are significantly below the lethal dose for normal cells, indicating that ivermectin is a safe and effective therapeutic candidate for human UC. Additionally, intravesical injection, which may increase the dose and effects of ivermectin, is a possibility for the treatment of non-muscle-invasive human UC in addition to oral and intravenous administration.

Ivermectin mediated cell cycle arrest at G1 phase was reported in human glioma, adult T cell leukemia/lymphoma, and canine mammary tumor cells 16-19. In this study, we also determined a G1 phase arrest in human UC cells under ivermectin treatment (Fig. 2A and 2B). Moreover, we demonstrated that ivermectin could induce caspase-dependent apoptosis in human UC cells. This finding is consistent with that of previous studies that used ivermectin to treat leukemia, glioblastoma, cad cervical, and colorectal cancer cells 7, 17, 20-23. However, it could not induce cellular apoptosis in human breast cancer cells 24, suggesting that ivermectin-induced apoptosis is not a general effect in human cancers. In addition, our results demonstrated that ivermectin activated both intrinsic and extrinsic caspase pathways to cause cellular apoptosis (Figures. 3 and 4). Moreover, the upstream pathways involved in ivermectin-mediated cellular apoptosis, including oxidative stress, NF-κB, or Akt/mTOR pathways have been previously reported 6, 21, 22. Importantly, in this study, we found a novel upstream mechanism, i.e., the JNK signaling pathway, involved in ivermectin-mediated apoptosis in UC cells (Figure. 5). In addition, activation of the Akt signaling pathway in RT4 cells decreased with ivermectin treatment (Figure. 5). Because ivermectin suppresses the Akt/mTOR pathway and further mediates apoptosis induction in ovarian cancer cells 21, we speculated that ivermectin can also regulate cellular apoptosis in human UC cells by inhibiting the Akt signaling pathway. However, whether oxidative stress or Akt/mTOR pathways are involved in ivermectin-mediated apoptosis in human UC cells needs further investigation. In addition, treatment with ivermectin can reduce phospho-Erk expression as well as apoptosis induction in RT4 cells (Figure 5A). However, our result demonstrated that suppression Erk pathway with PD98059 can't elevate cell apoptosis in RT4 cells under ivermectin treatment (Figure 5B). Therefore, Erk pathway is not involve in ivermectin mediated apoptosis in RT4 cells. Interestingly, suppression of Erk activity with PD98059 could reverse ivermectin mediated PARP activation and partially reducing cell apoptosis in RT4 cells, suggesting that suppression of Erk pathway plays a feedback regulation of ivermectin induced cell apoptosis. Further study needs to investigate this hypothesis.

Moreover, cisplatin-based regimens are used to treat patients with advanced stage UC; however, resistance and recurrence are common. Interestingly, ivermectin can synergize with chemotherapeutic agents including cisplatin and 5-fluorouracil to promote anticancer activity in esophageal squamous cell carcinoma cell 25, or with tamoxifen to treat breast and osteosarcoma 26. In addition, ivermectin shows synergistic activity with docetaxel, tamoxifen, and cyclophosphamide in breast and prostate cancers 24. These findings suggest that ivermectin could be combined with standard clinical therapeutic agents to treat certain types of human cancers. The possible synergistic effects of ivermectin with anti-UC chemotherapeutics agents require further investigation.

## Supplementary Material

Supplementary figures.
