# Supplementary material for: Ivermectin induces cell cycle arrest and caspase-dependent apoptosis in human urothelial carcinoma cells
Source: Int J Med Sci. 2022 Sep 11;19(10):1567–75. doi: 10.7150/ijms.76623 (PMC9515697; doi:10.7150/ijms.76623)
Supplement: Supplementary file 1 — Supplementary figures. [file ijmsv19p1567s1.pdf]

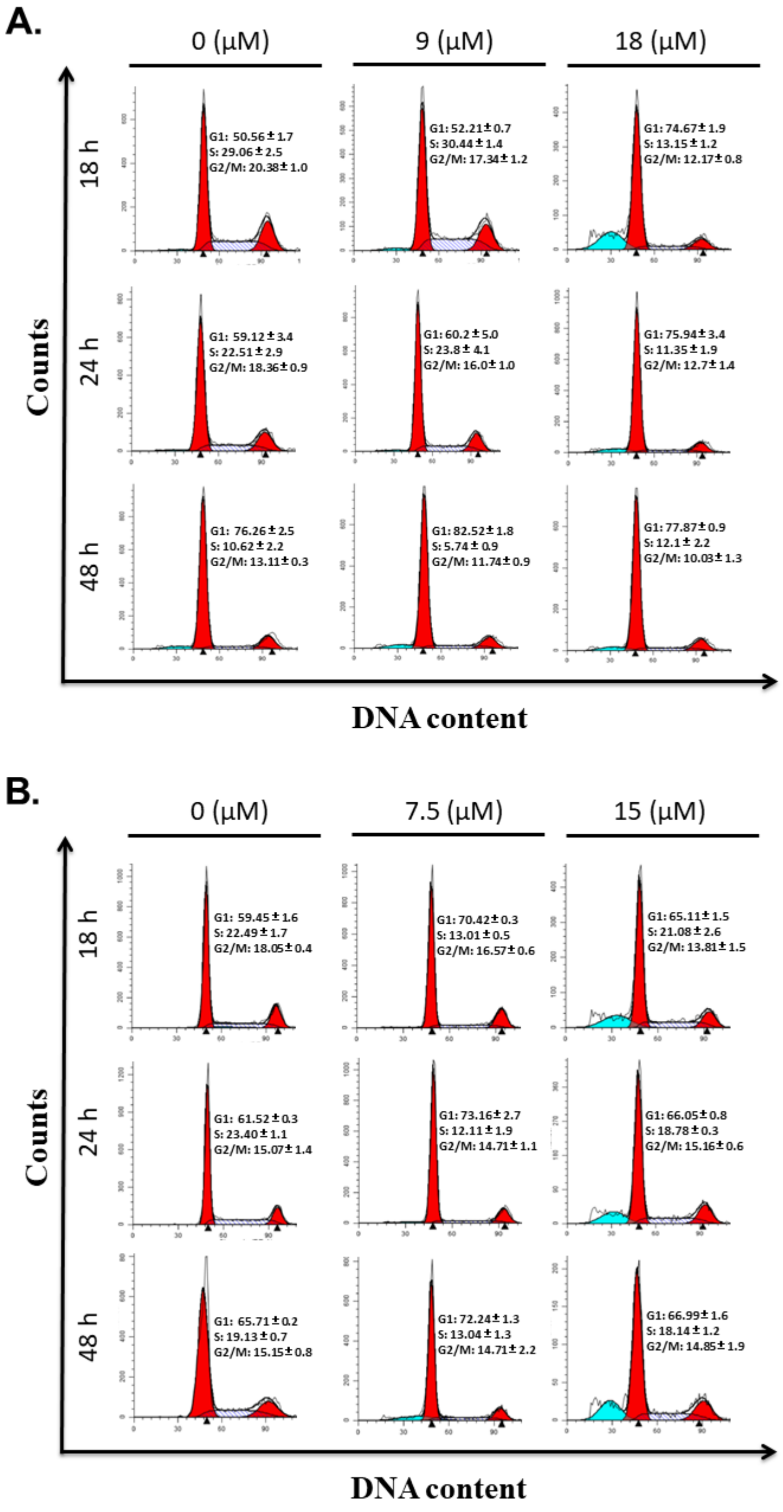

Supplementary Figure 1. Ivermectin induces cell cycle arrest in human UC cells. (A) T24 and (B) RT4 cells were treated with ivermectin, and the cell cycle phase was determined by flow cytometry. DMSO was used as the negative control.

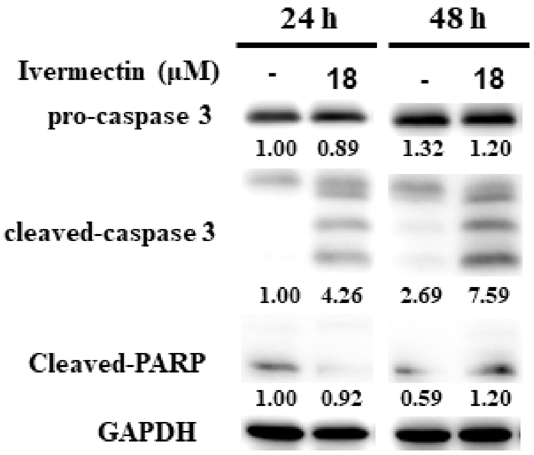

Supplementary Figure 2. Ivermectin induces cellular apoptosis in T24 cells. T24 cells were treated with ivermectin, and caspase-3 and PARP expression were assessed using western blotting. DMSO was used as negative control. Three independent western blotting experiments were conducted.
